# Regulation of Papillary Muscle Contractility by NAD and Ammonia Interplay: Contribution of Ion Channels and Exchangers

**DOI:** 10.3390/membranes12121239

**Published:** 2022-12-07

**Authors:** Alexey S. Averin, Maxim V. Konakov, Oleg Y. Pimenov, Miliausha H. Galimova, Alexey V. Berezhnov, Miroslav N. Nenov, Vladimir V. Dynnik

**Affiliations:** 1 Institute of Theoretical and Experimental Biophysics, the Russian Academy of Sciences, Pushchino 142290, Russia; 2 Institute of Cell Biophysics, the Russian Academy of Sciences, Pushchino 142290, Russia

**Keywords:** cardiomyocytes, papillary muscle, NAD/ammonia antagonism, contractility, electrophysiological characteristics, Kir2.x channels, HCN channels, Ca^2+^-activated K^+^ channels, NCX exchanger, K^+^-dependent positive/negative feedback loops

## Abstract

Various models, including stem cells derived and isolated cardiomyocytes with overexpressed channels, are utilized to analyze the functional interplay of diverse ion currents involved in cardiac automaticity and excitation–contraction coupling control. Here, we used β-NAD and ammonia, known hyperpolarizing and depolarizing agents, respectively, and applied inhibitory analysis to reveal the interplay of several ion channels implicated in rat papillary muscle contractility control. We demonstrated that: 4 mM β-NAD, having no strong impact on resting membrane potential (RMP) and action potential duration (APD90) of ventricular cardiomyocytes, evoked significant suppression of isometric force (F) of paced papillary muscle. Reactive blue 2 restored F to control values, suggesting the involvement of P2Y-receptor-dependent signaling in β-NAD effects. Meantime, 5 mM NH_4_Cl did not show any effect on F of papillary muscle but resulted in significant RMP depolarization, APD90 shortening, and a rightward shift of I–V relationship for total steady state currents in cardiomyocytes. Paradoxically, NH_4_Cl, being added after β-NAD and having no effect on RMP, APD, and I–V curve, recovered F to the control values, indicating β-NAD/ammonia antagonism. Blocking of HCN, Kir2.x, and L-type calcium channels, Ca^2+^-activated K^+^ channels (SK, IK, and BK), or NCX exchanger reverse mode prevented this effect, indicating consistent cooperation of all currents mediated by these channels and NCX. We suggest that the activation of Kir2.x and HCN channels by extracellular K^+^, that creates positive and negative feedback, and known ammonia and K^+^ resemblance, may provide conditions required for the activation of all the chain of channels involved in the interplay. Here, we present a mechanistic model describing an interplay of channels and second messengers, which may explain discovered antagonism of β-NAD and ammonia on rat papillary muscle contractile activity.

## 1. Introduction

Ion channels’ overexpression and knockout models, the models of heart failure and hypertrophy, are widely applied to investigate functional relations of multiple ion channel currents involved in the control of action potential (AP) waveform and duration, excitation–contraction coupling and/or cardiac automaticity using stem cells derived and isolated cardiac myocytes (CM), myocardial strips, and isolated hearts [1,2,3,4,5,6,7,8,9,10]. Numerous studies have demonstrated: (i) molecular coupling of voltage-dependent L-type calcium channels (LTCC, I_Ca-L_ current), ryanodine receptors (RyR)-mediated Ca^2+^-induced Ca^2+^ release (CICR; I_SR_), and small-conductance Ca^2+^-activated potassium channels (SK1-3 channels; I_SK_) being involved into repolarization and action potential (AP) waveform control (phases 2, 3 of AP) [1,2,3]; (ii) adjustment of fine balance of outward/inward rectification carried out, in turn, by both intermediate conductance Ca^2+^-activated K^+^ channels current (SK4 channels, I_IK_ current) and pacemaker “funny” current (I_f_) mediated by hyperpolarization-activated cyclic nucleotide-gated (HCN) channels, i.e., tight channels interplay (“I_IK_-induced I_f_ activation” effect, phase 3 of AP) involved in the automaticity in stem cell derived or ventricular CM with overexpressed SK4 and HCN channels [4,5]; (iii) synergistic effects of inward “funny” current I_f_ and outward K^+^ current (IK1_out_) mediated by strong inward rectifier K^+^ channels (Kir2.1), i.e., the effect contributing to the mechanisms of automaticity (“IK1_out_-induced I_f_ activation” effect, phase 3 of AP) [6]; (iv) cooperative interaction between inward K^+^ current (IK1_in_) mediated by Kir2.1 channels, and voltage-dependent Na^+^ current (Na_V_1.5, I_Na_) both contributing to the control of excitability (phase 4 of AP) [7]; (v) cooperation between I_f_, IK1_in_ + IK1_out_, I_Na_, and I_Ca_ delivering N-shaped current–voltage (I/V) relationship, which, in turn, underlies bistability or rhythmicity of the membrane potential [8]; (vi) dramatic alterations in the excitation–contraction coupling and Ca^2+^ homeostasis in transgenic mice overexpressing cardiac HCN channels characterized by shortening of AP duration and 3–4 fold increase of Ca^2+^ transients (CaT), based on the interplay of overactivated “funny” current I_f_ and Na^+^/Ca^2+^ exchanger reverse mode current (NCX, I_NCXrev_) driven by high [Na^+^]_I_ (phase 4 of AP) [9]; (vii) similar to the previous one the effect provided by the interplay of persistent L-type Ca^2+^ current (I_Ca-L_) and I_NCXrev_ in the cells with overexpressed LTCC and potassium channels [10].

However, compared to the studies on atrial CM, there is sparse information on the functional recruitment of multiple ion currents in the ventricular CM of working myocardium, and the available data are contradictory [2]. It is well recognized that Ca^2+^-activated K^+^ currents share an important feedback control of AP waveform and duration (APD) by coupling the increase in [Ca^2+^]_i_ to hyperpolarization of the plasma membrane (early and late repolarization, phases 2 and 3 of AP) [11,12,13]. Though SK2 channels, having low expression compared to SK4 channels [12,13], are considered to be dormant in cells of working myocardium [1,3] and may contribute to the shortening of APD, only being activated by the agonists of β-adrenoreceptors or at cardiac hypertrophy via protein kinase A (PKA)-dependent phosphorylation [3]. Similarly, the involvement of high (big) conductance Ca^2+^-activated K^+^ currents (I_BK_) in the control of AP waveform is a matter of debate due to the low expression of plasmalemmal BK channels in cardiac cells [11,12,13,14,15]. Most attention is focused on mitochondrial BK channels [16], which cannot be involved in the excitation–contraction coupling. Although, both impermeable (iberiotoxin) and permeable (paxilline) blockers of BK channels have a similar effect, by slowing the heart rate and increasing APD, indicating the possible involvement of plasmalemmal type I fast-gated BK channels in AP control [14,15]. The depolarizing “funny” current I_f_, which has an important role in the control of excitability by being involved in the adjustment of the depolarization phase in pacemaker cells (phase 4 of AP) [17], is often underestimated as a significant player in the cells of working myocardium, where it, nevertheless, may contribute to late repolarization and AP shape control [18].

Here, we applied β-NAD (NAD) and ammonia (NH_3_ + NH_4_^+^) in the form of NH_4_Cl, known hyperpolarizing and depolarizing agents, respectively, to reveal the implication of the above-mentioned cardiac ion channels in the control of rat papillary muscle’s (PM) contractility function. Both agents were selected owing to their multimodal direct/indirect effects on various ion channels, transporters, and second messengers signaling systems.

Extracellular NAD_o_ has been demonstrated to suppress electrical activity and contractility of gastrointestinal (GI) smooth muscle cells [19,20] and contraction of isolated rat aorta and arteries [21] and to promote the endothelial cell barrier integrity via a PKA-dependent mechanism [22].

Numerous studies have shown that NAD_o_ is a P2Y1 and P2Y11 purinoreceptors agonist that causes accumulation in various cell types of second messengers such as inositol-3-phosphate (IP3), cyclic ADP-ribose (cADPR), NAADP, cAMP, and Ca^2+^, assuming simultaneous implication of several signaling pathways coupled to phospholipase C (PLC), adenylate cyclase (AC), and ADP-ribosyl cyclase (ARC) [22,23,24,25,26]. At the present time, NAD_o_ is considered to be a purinergic inhibitory motor neurotransmitter involved in the control of GI smooth muscle cells motility, which evokes profound hyperpolarization of GI cells by recruiting the following signaling axis:

P2Y_1_ receptors/G_q_ proteins/PLCβ/IP3/IP3 receptors (IP3R)/Ca^2+^ (1)

which, in turn, activates SK2 channels mediating hyperpolarizing I_SK_ currents [20].

Compared to GI cells, the stimulation of P2Y_1_ receptors in rat striatal neurons recruits both BK and SK channels that mediate hyperpolarizing outward K^+^ currents responsible for the decrease of the frequency of neuronal firing [26].

In rodent hearts and cell preparations, NAD_o_ (i) slowed heart rate and cell automaticity; (ii) shortened APD of pacemaker cells and cells of working myocardium [27,28]; (iii) hyperpolarized membrane resting potential; and (iv) diminished the slope of diastolic depolarization (phase 4 of AP) in the preparations of atrial cells [27].

In turn, ammonia is an important metabolite and well-known neurotoxin [29,30,31,32]. Hydrated ions NH_4_^+^ and K^+^ have a similar ionic radius and, therefore, NH_4_^+^ competes with K^+^ at multiple channels and transporters [33], including Kir1.1 [34], Kir2.1 [35], Kir4.1 [36,37], SK2 [38,39], and HCN2 [40,41,42] channels, and Na^+^/K^+^-ATPase (NKA), and Na^+^/K^+^/2Cl^−^ co-transporters (NKCC) [37,43,44], etc. In addition, NH_4_^+^ activates BK channels by increasing Ca^2+^ affinity and evokes a leftward shift of the BK channels’ voltage dependence [45].

NH_4_Cl dose-dependently depolarizes the resting membrane potential of neurons [37,46,47] and astrocytes [37,47,48]. At concentrations higher than 5–6 mM, NH_4_Cl evoked robust activation of neuronal networks grown on microelectrode arrays [49] and induced neuronal networks’ burst firing in hippocampal neuronal and astrocyte co-cultures [50]. Networks overexcitation was suppressed by the blockers of NMDA and AMPA receptors [50], methyl-l-methionine (Vitamin U), or the blockers of HCN channels and NAD [51].

Thus, to summarize, NAD and ammonia show opposite “antagonizing” effects on cellular electrical properties acting as hyperpolarizing and depolarizing agents that affect cellular electrogenic function via direct and indirect modulation of multiple ion channels and exchangers. In the present study, we used these two physiologically active compounds to investigate the involvement of different ion channels and exchangers in the regulation of cardiac contractility. To investigate the implication of various channels in NAD/ammonia antagonism, we applied pharmacological inhibitory analysis and monitored the force of PM, the amplitude of which may correlate with calcium transient’s (CaT) amplitude [52,53,54]. Furthermore, we registered the resting membrane potential (RMP), action potential duration (APD90), and steady-state current–voltage (I–V) relationships of the total net current in isolated rat ventricular CM. Based on all these results, we discussed a mechanistic model describing channels and second messengers’ interplay, which may underlie the control of rat myocardial contractions under NAD_o_ and ammonia antagonism.

## 2. Materials and Methods

### 2.1. Animal Handling

Adult male Wistar rats (200–220 g body weight) were used for the experiments. This study did not involve endangered or protected species and was performed in accordance with Directive 2010/63/EU of the European Parliament. All experimental procedures were approved by the Biological Safety and Ethics Committee of the Institute of Cell Biophysics and the Institute of Theoretical and Experimental Biophysics.

### 2.2. Contractility of Papillary Muscles

Isolation of right ventricle papillary muscles was performed from the hearts of anesthetized rats. Measurements of the isometric force of PM contraction were performed in oxygenated (95% O_2_/5% CO_2_) Tyrode solution containing (in mM): NaCl, 135; KCl, 4; MgCl_2_, 1; CaCl_2_, 1.8; NaHCO_3_, 13.2; NaH_2_PO_4_, 1.8; glucose, 11; (pH 7.4), as previously described [55]. In brief, isolated PMs were mounted horizontally in a temperature-controlled chamber (30 ± 0.1°C) and stretched to a length at which the tension of contraction was maximal. Stimuli were applied using bipolar Ag–AgCl electrodes by square-wave pulses of 5 ms duration and amplitude set at 25% above the excitation threshold. Prior to each experiment, muscle preparations were stimulated at 0.3 Hz for 1 h until complete mechanical stabilization was achieved. The following parameters were recorded: the force of contraction, force–frequency relationship from 0.003 to 3 Hz, time to peak tension, and time relaxation to 50 and 90%. Here, we selected appropriate concentrations of various blockers based on their marked effect on the steady-state force of isometric contractions in control (up to 10–30%). PM preparations with a spontaneous or evoked time-dependent decline of the contraction (loss of steady state regimes) were removed.

### 2.3. Acute Isolation of Ventricular Cardiomyocytes 

Cardiomyocytes were isolated by enzymatic dissociation from the left ventricle of Wistar rats. After animal decapitation, the heart was extracted and retrogradely perfused for 3–5 min with DMEM + 10 mM HEPES medium (pH 7.25) (Sigma-Aldrich, MO, USA). After stabilization of cardiac contractions, perfusion was continued with a basic medium containing (in mM): NaCl, 80; KCl, 10; KH_2_PO_4_, 1.2; MgSO_4_, 5; glucose, 20; taurine, 50; HEPES, 10; l-arginine, 1, pH 7.25, supplemented with 2.5 mM EGTA, which was replaced by 20 mg/100 mL of protease Type XIV (Sigma-Aldrich, MO, USA), 100 mg bovine serum albumin (fraction V, Sigma-Aldrich, MO, USA), and 140 μM CaCl_2_ following cardiac arrest. After 10 min, the left ventricle was separated from the atria and right ventricle and cut into small fragments in a basic medium enriched with 200 μM CaCl_2_. Single cells were then isolated by stirring in the basic medium supplemented with protease Type XIV (Sigma) and collagenase IV (2.5 mg/10 mL; Worthington Biochemical Corp., NJ, USA) at 37 °C. The aliquots were removed at 20 min intervals until the tissue was entirely digested. Isolated cardiomyocytes were precipitated by centrifugation (600–800 rpm, 1 min), washed twice, and stored in basic medium containing 200 μM CaCl_2_ at room temperature.

### 2.4. Whole-Cell Patch Clamp Recordings

Raptured whole-cell patch clamp recordings from visually identified cardiomyocytes were performed using SliceScope (Scientifica, Uckfield, UK) equipped with a CCD camera. Recordings were done with PC505B amplifier (Warner Instruments, CT, USA) in voltage and current clamp modes. Signals were filtered at 2 kHz with the amplifier, then acquired and digitized at 10 kHz sampling frequency with Digidata 1440A (Molecular Devices, CA, USA) and software package for data acquisition and analysis pClamp 10.2 (Molecular Devices, CA, USA). Recording electrodes of 4–5 MOhm resistance were pulled from borosilicate glass capillaries (Harvard Apparatus, MA, USA) using a PC10 vertical puller (Narishige, NY, USA). Extracellular Hank’s solution contained (in mM): 139 NaCl, 4.2 NaHCO_3_, 0.4 NaH_2_PO_4_, 2.1 KCl, 0.44 KH_2_PO_4_, 1.25 CaCl_2_, 0.8 MgSO_4_, 4 HEPES, 8 D-glucose, pH 7.4, and osmolality 305 ± 2 mOsm. The composition of the intracellular solution was as follows (in mM): 120 K-gluconate, 3 KCl, 2 Na_2_ATP, 0.3 Na_2_GTP, 0.3 MgATP, 10 Na_2_-phosphocreatine, 1 MgCl_2_, 0.25 EGTA, 4 HEPES, pH 7.2 and osmolality of 280 ± 5 mOsm. All experiments were conducted at 30 °C. Access resistance was monitored throughout the recording and was typically < 35 MOhm.

### 2.5. Data Analysis and Statistics

Paired t-test was used to compare continuous variables. One-way ANOVA with Dunnett’s post hoc test was used for multiple groups comparison. *p*-value < 0.05 was predetermined as a statistically significant difference. All data are presented as mean ± standard error (S.E.).

## 3. Results

### 3.1. NAD and Ammonia Antagonism

#### 3.1.1. Effects of NAD on PM Contractions, RMP, and AP Duration of CM: P2Y Receptors Antagonist Abrogated Suppression of PM Contractions by NAD

**Figure 1:**
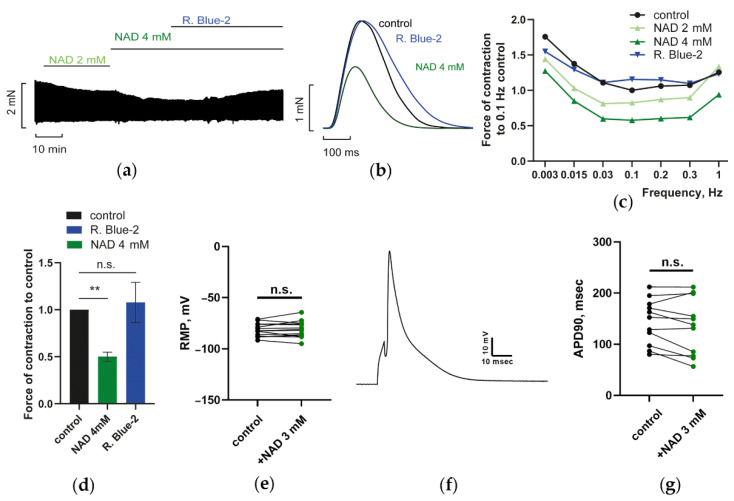
Suppression of PM contractions by NAD is abrogated by P2Y purinoreceptors antagonist Reactive Blue 2 (R. Blue 2). The impact of NAD on RMP and APD of isolated CM. (**a**) Representative trace of isometric contraction force F of the right ventricle papillary muscle (PM) characterizing dose-dependent suppression of force F by 2 and 4 mM NAD and following prevention of this effect by 100 µM R. Blue 2. Horizontal green and blue lines indicate the time of NAD and R. Blue 2 application, respectively; stimulation frequency *f* = 0.3 Hz. (**b**) Representative force F transients, characterizing the impact of NAD and R. Blue 2, are shown as green and blue vs. control black lines, respectively; *f* = 0.3 Hz. (**c**) The panel shows a representative force–frequency relationship (F_MAX_/*f*), characterizing the impact of NAD and R. Blue 2. The ordinate shows the amplitude of isometric force F (F_MAX_), normalized to that obtained at *f* = 0.1 Hz in the control. The abscissa shows the stimulation frequency f in Hz. Black, light green, dark green, and dark blue lines describe the control experiment and the effects of 2 mM NAD, 4 mM NAD, and 4 mM NAD + 100 µM R. Blue 2 applications, respectively. The data was taken in steady-state conditions 20–25 min after the application of NAD or R. Blue 2. (**d**) Mean, normalized to control *f* = 0.3 Hz F_MAX_, values of F_MAX_ are presented at (**d**) as the bars. Black, dark green, and blue bars characterize the control and the effects of 4 mM NAD and 4 mM NAD + 100 µM R. Blue 2, respectively. For each bar, the number of experiments *n* = 3. Data presented as mean ± SE. ** *p* < 0.01 with ANOVA post hoc Dunnett’s test, (**e**,**f**). (**e**) At 5 mM, extracellular NAD did not affect resting membrane potential RMP (**f**,**g**). At 5 mM, extracellular NAD did not affect AP duration (APD90) in isolated ventricular CM. Panel (**f**) shows the representative evoked AP used for the assessment of APD90 (Panel (**g**)). The measurements of RMP and APD90 were performed with a ruptured whole-cell patch-clamp technique, as described in the Methods. *p* > 0.05 with paired t-test. *n* = 14 and 11 for Panels (**e**,**g**), respectively. Paired t-test. Here and further below the abbreviation n.s. stands for non-significant results (*p* > 0.05).

Figure 1a–d demonstrates the effects of extracellular NAD and P2Y purinoreceptors antagonist Reactive Blue 2 (R. Blue 2) on the isometric force of paced PM. Figure 1a displays the track of isometric force F transients registered at stimulation frequency f of 0.3 Hz. This panel shows that at concentrations 2 to 4 mM, NAD dose-dependently suppressed force. Note that the inhibition of contractions was developed by slowly attaining steady-state values of maximal force (F_MAX_) within 15 to 20 min (Figure 1a). Application of R. Blue 2 (100 µM) abrogated the effect of NAD, evoking the slow restoration of PM contractions to control values within similar time intervals. Representative force F transients, characterizing the impact of NAD and R. Blue 2, are presented in Figure 1b (green and blue vs. control black lines). 

Figure 1c describes the respective force–frequency (F_MAX_/*f*) relations registered in the control PM (black line) and after sequential application 2 and 4 mM NAD (in green) or 4 mM NAD + R. Blue 2 (in blue). The maximal inhibitory effect of 4 mM NAD was observed at frequency f of 0.3 Hz and reached 48–50% of F_MAX_. Rising f to 1 Hz increased F_MAX_ and diminished the inhibitory effect of NAD. Therefore, we selected *f* = 0.3 Hz as the basic stimulation frequency for statistical evaluation of the effects studied. Mean, normalized to control 0.3 Hz F_MAX_, values of F_MAX_ are presented in Figure 1d as the bars. 

The measurements of resting membrane potential (RMP) and AP duration at the 90% level of repolarization (APD90) in isolated ventricular CM, performed with ruptured whole-cell patch-clamp technique, have indicated that extracellular NAD did not evoke any statistically significant alterations of RMP (–81.7 ± 1.7 mV in control versus –81.12 ± 2.1 mV in the presence of NAD, *n* = 14; *p* > 0.05 with paired t-test; Figure 1e) and AP duration (APD90 144.4 ± 13.4 msec in control versus 134.5 ± 16.7 msec in the presence of NAD, *n* = 11; *p* > 0.05 with paired t-test; Figure 1f) what did not correspond to the strong suppression of force evoked by NAD in PM. Here, we might suggest that some variation in APD may reflect the heterogeneity of CM populations. Besides, these results may indicate that measured electrical parameters of isolated CM do not fully reflect the alterations of force in paced PM that were evoked by the application of NAD.

Note that the P2Y purinoreceptors antagonist R. Blue 2 canceled the suppressant effect of NAD on PM by restoring force to control values in the whole diapason of stimulation frequencies from 0.003 to 1 Hz (Figure 1c). Taken together, these results suggest that switching on of P2Y receptor-dependent second messengers signaling axes may underlie the strong inhibition of PM contractions evoked by NAD, while the weak effect of NAD on electrical parameters of isolated non-loaded CM may be associated with low Ca^2+^ turnover in not stretched CM.

#### 3.1.2. At 5 mM, Ammonia Depolarized RMP and Shortened APD90 in CM, but Does Not Have Any Impact on PM Contractions

**Figure 2:**
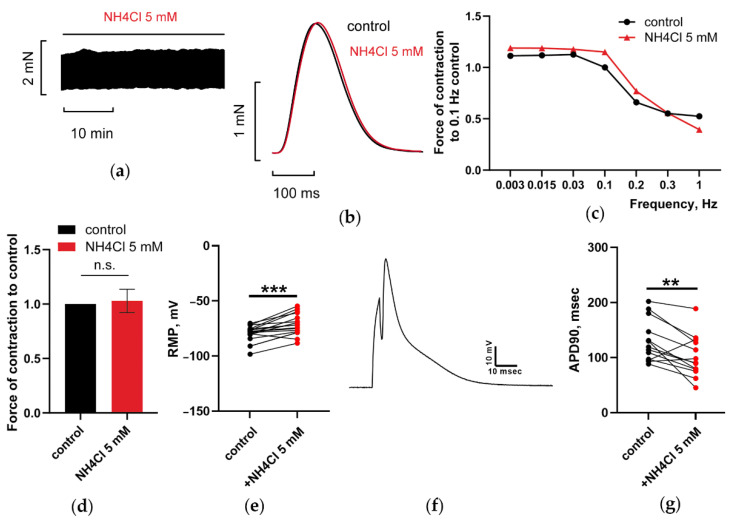
At 5 mM, NH_4_Cl does not have any impact on the isometric force of paced PM in spite of the depolarization of RMP and APD shortening in CM. (**a**,**b**) Representative traces of force and force transients of PM, respectively, recorded at a stimulation frequency *f* = 0.3 Hz. The effect of 5 mM NH_4_Cl is shown in red. (**c**) Representative force–frequency relationships (F_MAX_/*f*), characterizing the impact of 5 mM NH_4_Cl (red vs. black lines) and recorded at stimulation frequencies f ranging from 0.003 to 1 Hz, are shown in red. (**d**) Panel shows mean, normalized to control *f* = 0.3 Hz F_MAX_, values of F_MAX_ as the bars. The bars characterize the control (black) and the effects of 5 mM NH_4_Cl (red). *f* = 0.3 Hz. *p* > 0.05 with paired t-test. *n* = 3. (**e**) At 5 mM, NH_4_Cl evoked statistically significant depolarization of RMP, *n* = 17. Panel (**f**) shows the representative evoked AP used for the assessment of AP duration (APD90. (**g**) At 5 mM, NH_4_Cl evoked statistically significant shortening of APD90, in ventricular CM. *n* = 13. *** *p* < 0.005; ** *p* < 0.01 with paired t-test.

At concentrations up to 5–6 mM, NH_4_Cl does not have any effect on PM contraction at stimulation frequencies *f* ranging from 0.003 to 1 Hz (Figure 2a–d; red vs. black lines). 

However, as a well-known depolarizing agent [31], 5 mM NH_4_Cl significantly depolarized RMP of isolated CM from –79.8 ± 1.8 mV to −71.4 ± 2.3 mV (*n* = 17, *p* < 0.05 with paired t-test; Figure 2e). Contrary to the depolarization of RMP, ammonia evoked ~20% shortening of APD90 from 130.5 ± 10.6 msec to 101.4 ± 10.5 msec (*n* = 13, *p* < 0.05 with paired t-test, Figure 2g). Interestingly, the effect was statistically significant in spite of some level of variation of APD from cell to cell.

Here, we might speculate that the opposite impact of ammonia on RMP and APD, registered in isolated ventricular CM, may underlie its weak effect on the contractility of paced PM (Figure 2a–d).

#### 3.1.3. NH_4_Cl Restored Force of PM Contractions Suppressed by NAD despite Minor Alterations in RMP and APD of CM

**Figure 3:**
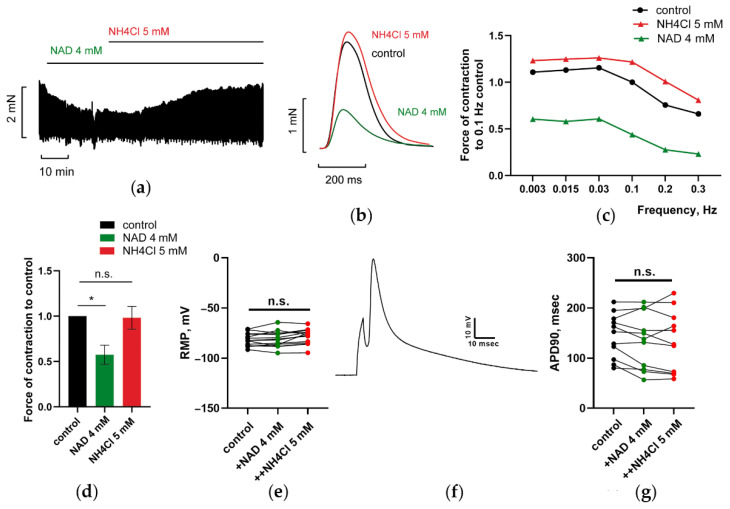
NAD and ammonia antagonism. At 5 mM, NH_4_Cl restores PM contractions suppressed by 4 mM NAD but does not induce substantial alterations in RPM and APD of CM. (**a**,**b**) Representative traces of force and force transients of PM, respectively, recorded at a stimulation frequency of *f* = 0.3 Hz. (**c**) Representative force–frequency relationships (F_MAX_/*f*), characterizing the impact of sequential application of 4 mM NAD and 5 mM NH_4_Cl. (**d**) Mean, normalized to control *f* = 0.3 Hz F_MAX_, values of F_MAX_ are presented at Panel (**d**) as the bars. * *p* < 0.05. *n* = 3 with ANOVA post hoc Dunnett’s test. In panels (**a**–**d**), the effects of NAD and NH_4_Cl are shown in green and red colors vs. black control. (**e**) 4 mM NAD canceled the effect of 5 mM NH_4_Cl on the electrical functions of CM-preventing induced by NH_4_Cl depolarization of RPM, *n* = 14. (**f**,**g**) 4 mM NAD canceled the effect of 5 mM NH_4_Cl on the electrical functions of CM, preventing induced by NH_4_Cl shortening APD90 in ventricular CM, *n* = 11. (**f**) show representative evoked AP used for assessment of APD90 (Panel (**g**)). *p* > 0.05 with ANOVA post hoc Dunnett’s test.

At a concentration of 5 mM, NH_4_Cl abrogated the inhibitory effect of 4 mM NAD on PM contractility by increasing force to or over control values (Figure 3a–d, red vs. green lines). Paradoxically, the restoration of force transients by ammonia in PM was not correlated with minor alterations in RMP and APD in CM after the combined application of NAD and ammonia. Figure 3e–g demonstrates that pretreatment of CM with 4 mM NAD prevented the expected marked depolarization of RMP (–81.7 ± 1.7 mV in the control versus –78.5 ± 2 in the presence of NAD and NH_4_Cl, *n* = 14, *p* > 0.05 with paired t-test) and APD90 shortening (144.4 ± 13.4 msec versus 132.7 ± 18.3 msec, *n* = 11, *p* > 0.05 with paired t-test) by NH_4_Cl in most of cells.

Here, we ought to outline that our populations of isolated CM include both epi-and endocardial CM subtypes, which are known to be characterized by distinct contractile and electric parameters [56], which may underlie the heterogeneity of electrical responses of individual cells (Figure 1e–g, Figure 2e–g and Figure 3e–g). 

Certainly, the slow development of the effects of NH_4_Cl and NAD on the force of paced PM, may suggest the possible involvement of second messengers and respective protein kinases in the regulation of various ion channels and contractile proteins involved in AP shaping, ECC control, and NAD/ammonia antagonism. In respect to this, we might suppose that such integral parameters, as RMP and APD, characterizing electrical functions of isolated resting CM, could not be taken as key comparison parameters characterizing contractile functions of stretched and paced PM at NAD/ammonia interplay.

Presented below, the analysis of current–voltage (I–V) relations may help to shed some light on this problem.

#### 3.1.4. Impact of NH_4_Cl, NAD, and K^+^ on Steady-State Current–Voltage (I–V) Relations of Net Current during Repolarizing Voltage Steps in Ventricular CM

**Figure 4:**
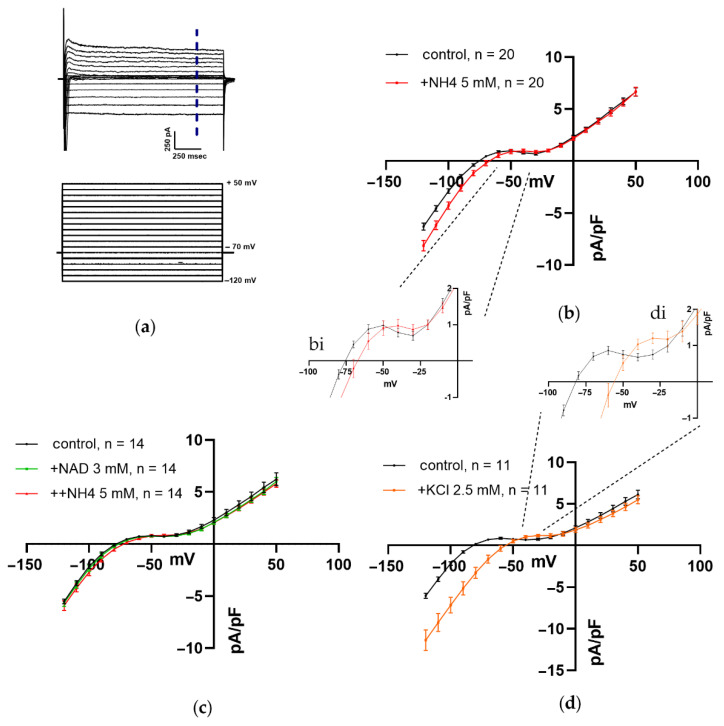
Effects of NH_4_Cl, NAD, and KCl on steady-state current–voltage (I–V) relationships of total net current in ventricular CM. NH_4_Cl and KCl evoked a rightward shift of I–V relations ((**b**), inset bi, (**d**), inset di), while NAD canceled the depolarizing effect of NH_4_Cl (**c**). (**a**) The panel shows voltage and current protocol. The currents were evoked by 10 mV voltage step between -120 mV to + 50 mV from a holding potential of −70 mV. Net currents were measured at 1.5 s (dashed line) using the illustrated pulse protocol. (**b**–**d**) The panels show I–V relations, characterizing the effects of added 5 mM NH_4_Cl (panel (**b**), red vs. black control, *n* = 20), sequential application of 4 mM NAD and 5 mM NH_4_Cl (panel (**c**)), green and red lines vs. black control, *n* = 154, and KCl (panel (**d**), 5.3 mM total vs. 2.8 mM in control, brown vs. black lines, *n* = 11), respectively. The panel insets bi and di represent enlarged windows of panels (**b**) and (**d**), respectively. Other details are in the text.

I–V relations, characterizing integral (net) sustained (steady-state) inward and outward currents, are illustrated in Figure 4. Both arms of the net current were evoked in CM held at −70 mV by a 10 mV voltage step increment from −120 mV to + 50 mV. Representative traces of recorded currents, along with the stimulation protocol, are shown in Figure 4a. Average current density at I–V relations was expressed as absolute current values normalized to the cell capacitance (Figure 4b–d).

The rectification of sustained currents was registered 1.5 s after the application of voltage steps to cut off instantaneous and fast transient inward and outward currents. Here, we suggested that the currents registered during the repolarizing voltage steps and contributing to net current were mostly mediated by Kir2.x, HCN, either-go-go and two-pore-domain (K2P) potassium channels.

Figure 4b–d displays the impact of NH_4_Cl, NAD + NH_4_Cl, and extracellular K^+^ on current–voltage relationships, respectively. Insets bi and di represent an enlarged window of I–V_m_ relationships presented in Figure 4b–d, respectively. The application of 5 mM NH_4_Cl, as shown in Figure 4b, evoked potentiation of sustained inward current (for example, at voltage step of −120 mV it was −6.5 ± 0.5 pA/pF in control versus −8.5 ± 0.6 pA/pF in the presence of NH_4_Cl, *n* = 14, *p* < 0.05 with paired t-test) and rightward shift I–V curve that corresponds to inward rectifying fraction of integral current in the depolarization direction decreasing reversion potential Er from ~−75 mV to −65.8 mV. Although the increase of the hump-like component of outward current over control values was statistically insignificant (enlarged part of I–V curve presented at Insert b), there was a concomitant rightward shift in the potential at which the hump-like component of the integral current reached its peak current density (from −50 mV in control to −40 mV in the presence of NH_4_Cl). Notably, pretreatment of CM with 4 mM NAD prevented alterations in I–V relationships induced by NH_4_Cl (Figure 4c), indicating NAD/ammonia antagonism similar to that one registered for RMP and APD (Figure 3e,f).

Previous studies have shown that the strong dependence of Kir2.x-mediated currents on extracellular K^+^ [57,58,59,60,61] may provide N-shaped hump-like I–V relationships and a strong rightward shift of Er, attained at high [K^+^]_o_ [58,60,61].

Figure 4d and Insert d both demonstrate that the increase of [K^+^]_o_ from 2.8 mM (control) to 5.3 mM ensures a rightward shift of inward rectifying current resulting in depolarization of Er by ~25 mV along with a strong rightward shift (~30 mV) for the voltage at which hump-like component of outward current reaches its maximum suggesting the implication of Kir2.x–mediated current (IK1) in this effect and underpinning close resemblance of potassium and ammonium ions (compare Inserts b and d). 

Taking together, we may suggest that Kir2.x-mediated current (IK1) may be involved in NAD/ammonia antagonism and PM contractility control.

### 3.2. Involvement of Kir2.x and HCN Channels, and Reverse Mode Na^+^-Ca^2+^ Exchanger in NAD and Ammonia Antagonism in Paced PM

#### 3.2.1. Blockade of Kir2.x Channels Prevents NH_4_Cl Effect

**Figure 5:**
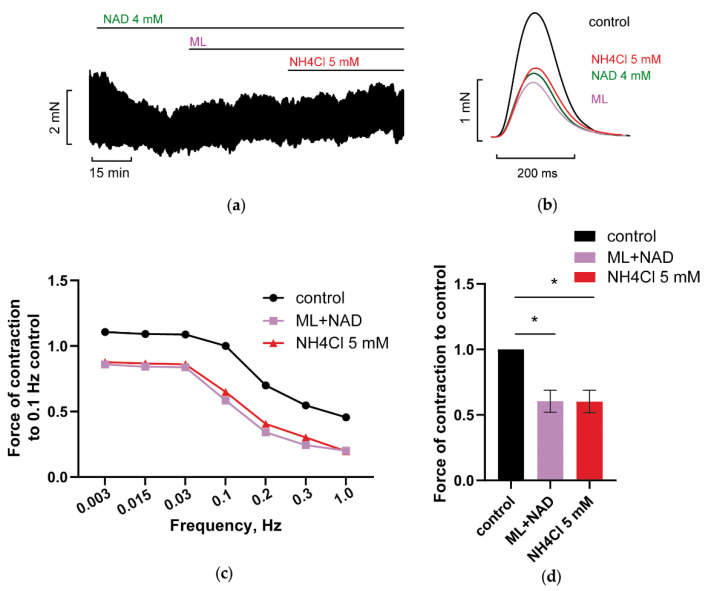
Kir2.x channels blocker ML133 abrogates NAD/ammonia antagonism abolishing restoration of PM contractility evoked by NH_4_Cl. (**a**–**d**) The panels demonstrate the blockade of Kir2.x channels with 10 µM ML133 prevented the restoration of PM contractions by 5 mM NH_4_Cl in PM preparations pretreated with 4 mM NAD. The effects of 4 mM NAD, 10 µM ML133 (ML), and 5 mM NH_4_Cl are shown in green, purple, and red, respectively. (**a**,**b**) Representative traces of force F and force F transients of PM, respectively, were recorded at stimulation frequency of *f* = 0.3 Hz. (**c**) Representative force–frequency relationships (F_MAX_/*f*) were recorded at stimulation frequencies f ranging from 0.003 to 1Hz. (**d**) Panel shows mean, normalized to control *f* = 0.3 Hz F_MAX_, values of F_MAX_ as the bars. *f* = 0.3 Hz. * *p* < 0.05 with ANOVA post hoc Dunnett’s test; *n* = 3.

Both arms of strong inward rectifier Kir2.x channels mediated IK1current are known to be involved in the setting of RMP and control of automaticity in excitable cells. The rightward shift of I–V_m_ relationships evoked by 5 Mm NH_4_Cl (Figure 4b) supports this notion. Collectively, we might suggest the implication of IK1_out_/IK1_in_ currents in NAD/ammonia interplay. Indeed, the blockade of Kir2.x channels with 10 µM ML133 prevented the restoration of PM contractions by 5 mM NH_4_Cl in PM preparations pretreated with 4 mM NAD (Figure 5; purple vs. red lines or bars) displaying the implication of Kir2.x channels in the control of PM contractions and NAD/ammonia antagonism. 

HCN channels, also being strongly activated by extracellular potassium ions [62,63,64,65], may cooperate with Kir.2.x channels, providing the interplay of outward Kir2.x mediated current (IK1_out_) and I_f_ current, known as “IK1_out_-induced I_f_ activation” effect in cardiac cells (realized at phase 3 of AP) [6]. Therefore, at this point, we may propose the involvement of HCN channels in NAD/ammonia antagonism.

#### 3.2.2. Blockade of HCN Channels Prevents NH_4_Cl Effect

**Figure 6:**
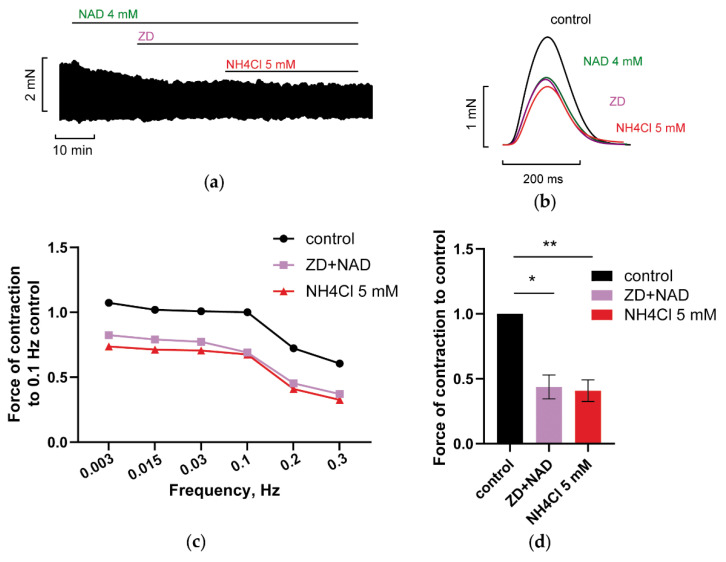
HCN channels blocker ZD 7288 prevents NAD/ammonia antagonism abolishing the restoration of PM contractility evoked by NH_4_Cl. (**a**–**d**) The panels demonstrate that the blockade of HCN channels with 20 µM Zd7288 (Zd) canceled the restoration of PM contractions by 5 mM NH_4_Cl in PM pretreated with 4 mM NAD. The effects of 4 mM NAD, 20 µM Zd7288 (ZD), and 5 mM NH_4_Cl are shown in green, purple, and red, respectively. (**a**,**b**) The panels show representative traces of force F and force F transients of PM, respectively, recorded at stimulation frequency *f* = 0.3 Hz. (**c**) Representative force–frequency relationships (F_MAX_/*f*) were recorded at stimulation frequencies f ranging from 0.003 to 0.3 Hz. (**d**) Panel shows mean, normalized to control *f* = 0.3 Hz F_MAX_, values of F_MAX_ as the bars. *f* = 0.3 Hz. * *p* < 0.05, ** *p* < 0.01 with ANOVA post hoc Dunnett test; *n* = 3.

“Funny” current, i.e., I_f_ is well recognized to control the automaticity of excitable cells [17]. However, some data suggest that this current may also contribute to APD control, being involved in phase 3 repolarization of AP in the cells of working myocardium [18]. Importantly, the upregulation of If current in epicardial cells may contribute to the prolongation of AP duration compared to endocardial cells [18]. Here, the application of HCN channels blocker ZD 7288 (20 µM) also abrogated NAD and ammonia antagonism (Figure 6a–c, green and purple vs. red lines; Figure 6d, purple vs. red bars), indicating the implication of the I_f_ current in NAD/ammonia interplay.

HCN channels, in turn, may cooperate with Na^+^-Ca^2+^ exchanger (NCX), reverse mode operation of which strongly depends on [Na^+^]_i_ [66,67].

#### 3.2.3. Inhibition of Reverse Mode Na^+^-Ca^2+^ Exchanger (NCX) Abolishes NAD and Ammonia Antagonism

It is well established that cardiac NCX exchanger is an important regulator of intracellular ion homeostasis. NCX is electrogenic transporter and the direction of its flux depends on Na^+^ and Ca^2+^ transmembrane gradients, what makes it difficult to predict its impact on AP. Presently, both the role of the Ca^2+^ entry and exit modes at physiological conditions during generation of AP are subjects of controversy [66,67,68]. Nevertheless, it is often considered that, at control conditions, NCX extrudes Ca^2+^ from ventricular myocytes, balancing the Ca^2+^ entering the cytoplasm through LTCC at AP. While some findings indicate that AP duration is strongly influenced by [Na^+^]_i_, with reverse mode NCX operation (2Ca^2+^ entry/3Na^+^ exit), i.e., providing repolarizing effect at high [Na^+^]_i_, that is enhanced in heart failure [66,69,70].

**Figure 7:**
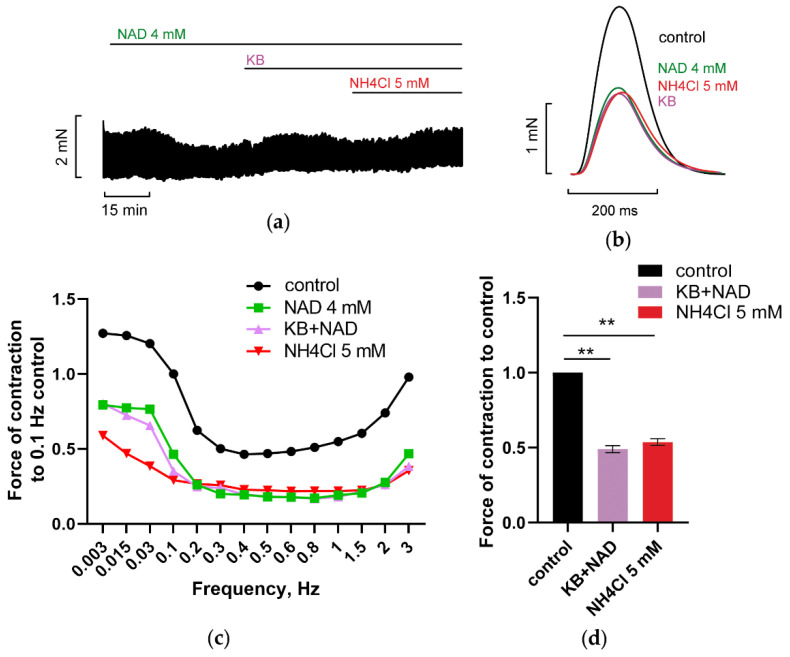
KB-R7943, the inhibitor of reverse mode NCX exchanger, prevents NAD/ammonia antagonism abolishing the restoration of PM contractility evoked by NH_4_Cl. (**a**–**d**) The panels demonstrate that the inhibition of reverse mode NCX exchanger by 10 µM KB-R7943 (KB) prevented the restoration of PM contractions by 5 mM NH_4_Cl in PM pretreated with 4 mM NAD. The effects of 4 mM NAD, 10 µM KB-R7943 (KB), and 5 mM NH4Cl are shown in green, purple, and red, respectively. (**a**,**b**) The panels show representative traces of force F and force F transients of PM, respectively, recorded at stimulation frequency *f* = 0.3 Hz. (**c**) Representative force–frequency relationships (F_MAX_/*f*), recorded at stimulation frequencies f ranging from 0.003 to 3 Hz. (**d**) Panel shows mean, normalized to control *f* = 0.3 Hz F_MAX_, values of F_MAX_ as the bars. *f* = 0.3 Hz. ** *p* < 0.01 with ANOVA post hoc Dunnett’s test ** *p* < 0.01; *n* = 3.

Here, we hypothesize that over activation of “funny” current I_f_, that may be realized at NAD/ammonia interplay, increased cytoplasmic [Na^+^]_i_ and raised driving force of reverse mode NCX, providing secondary increase SR and [Ca^2+^]_i_, that, in turn, resulted in the restoration of force F of PM. Figure 7 shows that the application of reverse mode NCX inhibitor KB-R7943 (10 µM) prevented NAD and ammonia antagonism (Figure 7a–c, purple vs. red lines; Figure 7d, purple vs. red bars), indicating the implication of reverse mode NCX-mediated current (I_NCXrev_) in NAD/ammonia interplay.

It is well known that depending on the experimental protocol, NCX isoforms, and species, NCX IC50 values for KB-R7943 are varied from dozens nM to dozens μM. Besides, in ventricular CM, KB-R7943 ma also inhibit I_Ca-L_ current and to lesser extent K_v_ currents [65]. To avoid overlapping effects, we selected 10 µM KB-R7943. Figure 7c shows that 10 µM KB-R7943 evoked a steep increase of isometric force with *f* rise from 1 to 3 Hz. Similar U-shaped F/*f* relationships are presented in Figure 1 and also were registered in the control experiments with KB-R7943 (without NAD). On the contrary, blockade of LTCC channels with nifedipine evoked a gradual fall of force with *f* rise up to 3 Hz (see 3.3., below), indicating the absence of overlapping effects of KB-R7943 on NCX exchanger and LTCC channels.

### 3.3. Blockade of Voltage-Gated L-Type Calcium Channels (LTCC) Abrogated NAD and Ammonia Antagonism

**Figure 8:**
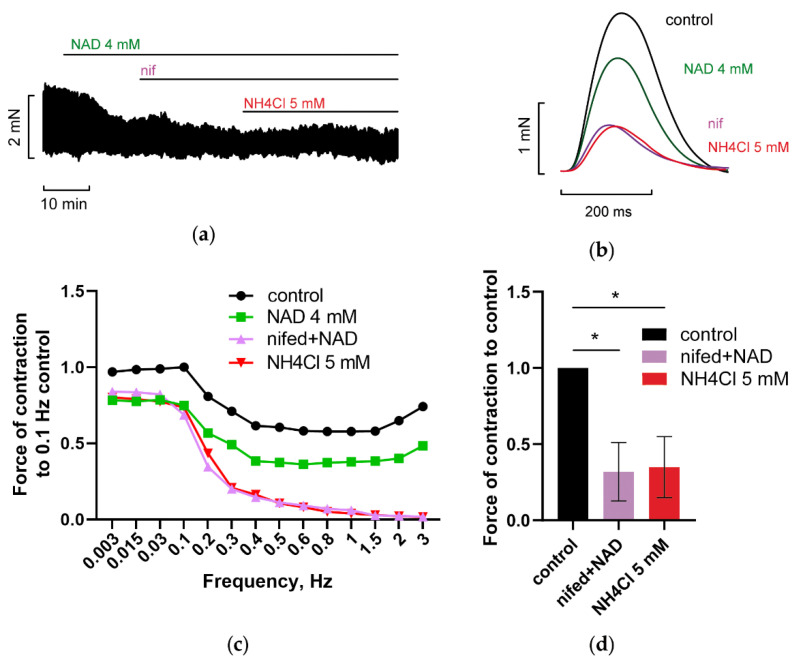
LTCC blocker nifedipine prevents NAD/ammonia antagonism abolishing the restoration of PM contractility evoked by NH_4_Cl. (**a**–**d**) The panels demonstrate that blockade of LTCC with 2 µM nifedipine (nif) abrogated the restoration of PM contractions by 5 mM NH_4_Cl in PM pretreated with 4 mM NAD. The effects of 4 mM NAD, 2 µM nifedipine (nif), and 5 mM NH_4_Cl, are shown in green, purple, and red, respectively. (**a**,**b**) The panels show representative traces of force F and force F transients of PM, respectively, recorded at stimulation frequency *f* = 0.3 Hz. (**c**) Representative force–frequency relationships (F_MAX_/*f*), recorded at stimulation frequencies f ranging from 0.003 to 3 Hz. (**d**) Panel shows mean, normalized to control *f* = 0.3 Hz F_MAX_, values of F_MAX_ as the bars. *f* = 0.3 Hz. * *p* < 0.05 with ANOVA post hoc Dunnett’s test; *n* = 3.

It is well known that the LTCC mediated I_Ca-L_ current control plateau phase of AP (phase 2 of AP) is involved in the ignition of calcium release (via CICR-dependent mechanism) from RyR-dependent stores and excitation–contraction coupling control [71]. Blockade of these channels, therefore, must ultimately suppress myocardial cell contractility independently of NAD and ammonia effects and prevent its interplay. Figure 8 demonstrates that blockade of LTCC by 2 µM nifedipine abolished recovery of PM contractility evoked by 5 mM NH_4_Cl in the presence of 4 mM NAD and abrogated NAD and ammonia antagonism. Note, that nifedipine induced a gradual fall of force with *f* rise, providing very low values of F at *f =* 2–3 Hz, which indicates well known strong dependence of F on I_Ca-L_ current at high values of stimulation frequency (Figure 8c). 

Besides, L-type Ca^2+^ current evokes two opposite effects on AP: (i) counteracts hyperpolarizing effects of K^+^ currents; (ii) induces a steep rise of [Ca^2+^]_i_ necessary for the activation of Ca^2+^-activated K^+^ channels supplying extracellular K^+^ required, in turn, for the activation of Kir.2 and HCN channels.

### 3.4. Implication of Calcium—Activated Potassium Channels in NAD and Ammonia Antagonism

Potentially, all three types of Ca^2+^-activated K^+^ channels (BK, IK, and SK) may be involved in the control of repolarization (2 and 3 phases of AP) and APD in CM of working myocardium providing redundant (robust) control of AP.

#### 3.4.1. Blockade of BK Channels Prevents NH_4_Cl Effect

**Figure 9:**
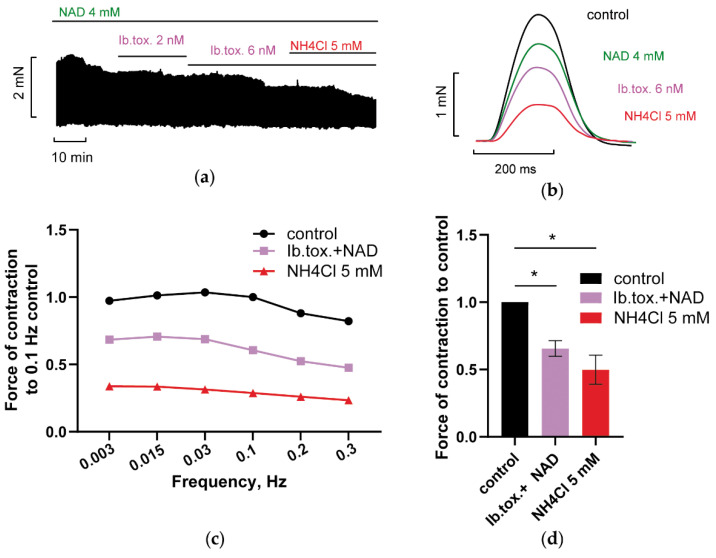
BK channel blocker Iberiotoxin abrogates NAD/ammonia antagonism preventing the restoration of PM contractility evoked by NH_4_Cl. (**a**–**d**) The panels demonstrate that the blockade of calcium-activated potassium BK channels with 6 nM iberiotoxin (Ib.tox.) prevented the restoration of PM contractions by 5 mM NH_4_Cl in PM pretreated with 4 mM NAD. The effects of 4 mM NAD, 6 nM iberiotoxin (Ib.tox.), and 5 mM NH_4_Cl are shown in green, purple, and red, respectively. (**a,b**) The panels show representative traces of force F and force F transients of PM, respectively, recorded at stimulation frequency *f* = 0.3 Hz. (**c**) Representative force–frequency relationships (F_MAX_/*f*), recorded at stimulation frequencies f ranging from 0.003 to 0.3 Hz. (**d**) Panel shows mean, normalized to control *f* = 0.3 Hz F_MAX_, values of F_MAX_ as the bars. *f* = 0.3 Hz. * *p* < 0.05 with ANOVA post hoc Dunnett’s test; *n* = 3.

The implication of plasmalemma big (large)—conductance BK channels in the control of myocardial contractility has been a matter of debate for several decades (see Introduction). However, in our experiments, application 6 nM of fast-gated type I BK channel blocker Iberiotoxin (Ib.tox., Figure 9a,b) reinforced the effect of NAD on force F, abolished NAD/ammonia antagonism, and even transformed the activating effect of ammonia to an inhibitory one (Figure 9b–d, purple vs. red lines and bars). Iberiotoxin is known to be an impermeable BK channels blocker, what might indicate that plasmalemma BK channels mediated current (I_BK_) may contribute to NAD and ammonia interplay. Note that Iberiotoxin was effective at, as a low concentration, 6 nM compared to usually applied values of 50 to 200 nM [14,15]. At 10–15 nM, Iberiotoxin strongly suppressed the force of PM contractions to 10–20% of the control.

#### 3.4.2. Blockade of IK Channels Prevents NH_4_Cl Effect

**Figure 10:**
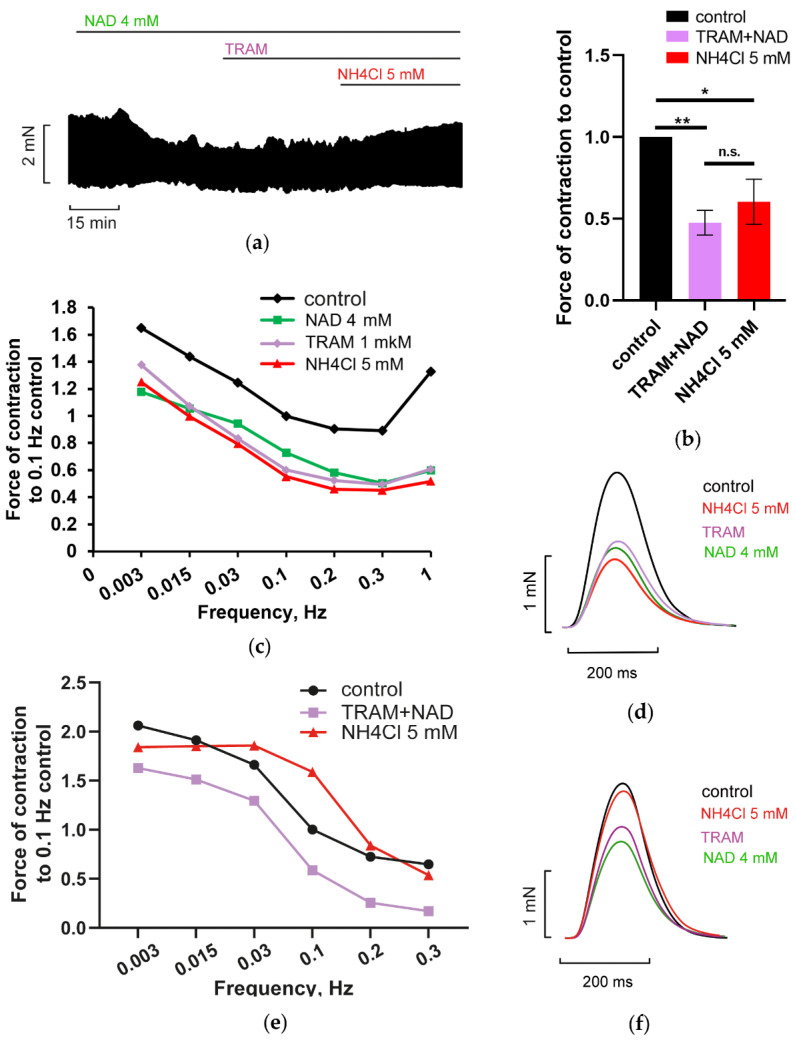
IK channels blocker TRAM 34 prevents NAD/ammonia antagonism abrogating restoration of PM contractility evoked by NH_4_Cl. (**a**–**f**) The panels demonstrate that the blockade of calcium-activated potassium IK channels by 1 µM TRAM 34 (TRAM) prevented the restoration of PM contractions by 5 mM NH_4_Cl in PM pretreated with 4 mM NAD. The effects of 4 mM NAD, 1 µM TRAM 34 (TRAM), and 5 mM NH_4_Cl are shown in green, purple, and red, respectively. (**a**,**d**,**f**) The panels show representative traces of force (**a**) and force transients of PM (**d,f**), respectively, recorded at stimulation frequency *f* = 0.3 Hz and characterize strong (**d**) and weak (**f**) effects of TRAM 34 on the force of PM contractions. Representative force–frequency relationships (F_MAX_/*f*), recorded at stimulation frequencies f ranging from 0.003 to 1 Hz, characterizing the strong impact of TRAM 34, are shown in panel (**c**). Representative traces of force transients and F_MAX_/*f* relationships, characterizing the weak impact of TRAM 34, are shown in panels (**e**). (**b**) The panel shows mean, normalized to control *f* = 0.3 Hz F_MAX_, values of F_MAX_ as the bars. *f* = 0.3 Hz. * *p* < 0.05, ** *p* < 0.01 with ANOVA post hoc Dunnett’s test; *n* = 5.

Intermediate conductance IK (SK4) channels are known as the most common of three types of Ca^2+^-activated K^+^ channels involved in AP shaping of excitable and working types of cardiac cells. Figure 10 shows that the application of IK channels blocker TRAM-34 (1 µM) abrogated NAD/ammonia antagonism, significantly preventing the complete restoration of PM contractility by 5 mM NH_4_Cl (green and purple vs. red lines or bars). This effect of TRAM 34 points to the involvement of IK channels mediated current (I_IK_) in NAD and ammonia interplay. Note, that the impact of TRAM 34 may vary from preparation to preparation (Figure 10c,d vs. Figure 10e,f). However, on average, the effect of 1 µM TRAM 34 is significant (Figure 10b). The application of 3 to 5 µM TRAM 34 may evoke non-specific effects. 

#### 3.4.3. Blockade of SK Channels Prevents NH_4_Cl Effect

**Figure 11:**
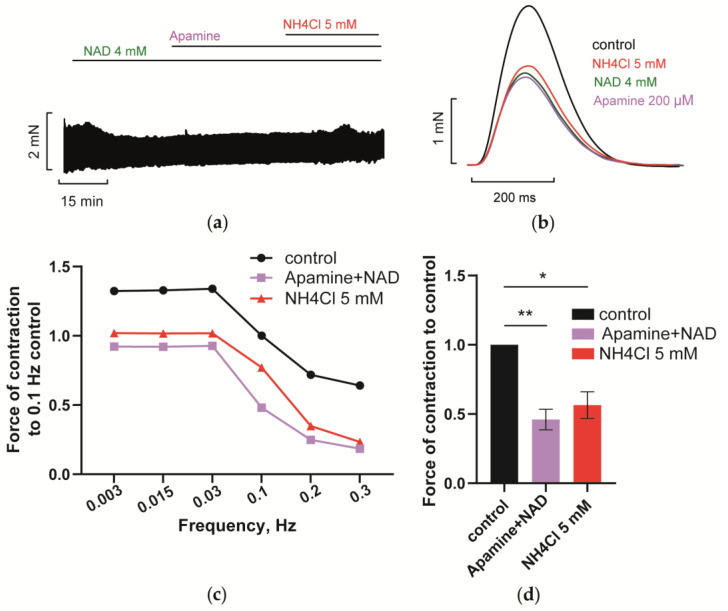
SK channels blocker apamin abrogates NAD/ammonia antagonism abolishing restoration of PM contractility evoked by NH_4_Cl. (**a**–**d**) The panels demonstrate that the blockade of calcium-activated potassium SK channels with 200 nM apamin prevented the restoration of PM contractions by 5 mM NH_4_Cl in PM pretreated with 4 mM NAD. The effects of 4 mM NAD, 200 nM apamin, and 5 mM NH_4_Cl are shown in green, purple, and red, respectively. (**a**,**b**) The panels show representative traces of force F and force F transients of PM, respectively, recorded at stimulation frequency *f* = 0.3 Hz. (**c**) Representative force–frequency relationships (F_MAX_/*f*) recorded at stimulation frequencies f ranging from 0.003 to 0.3 Hz. (**d**) Panel shows mean, normalized to control *f* = 0.3 Hz F_MAX_, values of F_MAX_ as the bars. *f* = 0.3 Hz. * *p* < 0.05, ** *p* < 0.01 with ANOVA post hoc Dunnett’s test; *n* = 5.

Ventricular slow-conductance SK (SK1-3) channels are considered to be dormant under control conditions and are activated in heart hypertrophy or increased adrenergic drive [1,2,3]. Figure 11 shows that apamin (200 nM), the blocker of SK channels, also abolished NAD/ammonia antagonism. Subsequent application of 5 mM NH_4_Cl did not restore PM contractility suppressed by 4 mM NAD (green and purple vs. red lines or bars). Apparently, the SK channel-mediated current (I_SK_) could contribute to NAD and ammonia interplay, as it might be activated by PKA via the P2Y_11_ receptors-dependent signaling axis [3,26].

Collectively, the results suggest consistent cooperation of IK1_out_/IK1_in_, I_f_, I_Ca-L_, I_SK_, I_IK_, I_BK_, and I_NCXrev_ currents in NAD/ammonia interplay.

## 4. Discussion

In this work, we apply the force of isometric PM contractions (F) as a final integral measure of NAD/ammonia effects. With some limitations, the amplitude of F may be used as a measure of CaT amplitude because the changes of CaT and force often correlate, except in the conditions of covalent control of contractile proteins evoked by β-adrenergic drive (ref. [53]). We might expect similar effects in NAD/ammonia interplay at the switching on of P2Y receptor signaling by NAD. Here, we have demonstrated that 5 mM NH_4_Cl induced recovery of rat PM contractions suppressed by NAD_o_ (Figure 3) by rising F_MAX_ to or over control values, indicating that restoration of CaT and excitation–contraction coupling by ammonia may depend on the impact of second messengers of several signaling pathways switched on by NAD_o_.

### 4.1. NAD and Second Messengers IP3, cAMP and cADPR

Presently, NAD_o_ is considered as a purinergic inhibitory motor neurotransmitter in GI smooth cell preparations that evokes hyperpolarization of GI cells by activating Ca^2+^-dependent SK2 channels via the signaling axis [20]:

P2Y_1_ receptors/G_q_ proteins/PLCβ/IP3/IP3 receptors (IP3R)/Ca^2+^ (1)

Besides, NAD is known to serve as a coenzyme for numerous cellular oxidation–reduction reactions, as a substrate of multiple deacetylases involved in posttranslational modification of proteins, and a substrate of extra- and intracellular NAD glycohydrolases CD38 (of ARC generating second messenger cADPR) [72].

In 1991, Lee et al. discovered that cADPR, synthetized from NAD, modulated Ca^2+^-induced Ca^2+^ release from ER stores in sea urchin eggs [73,74]. Since that time, the implication of cADPR in the facilitation of RyR- encoded CICR has been widely recognized [71]. In 2001, extracellular NAD_o_ was shown to induce RyR-dependent [Ca^2+^]_i_-overshoots and oscillations in hippocampal astrocytes [75]. The proposed model of auto-and paracrine control of [Ca^2+^]_i_-homeostasis suggested the release of NAD from astrocytes, its conversion to cADPR by extracellular CD38, and back import of cADPR into neurons and astrocytes with subsequent impact at RyR [76]. Soon after, it was demonstrated that various hormones and neurotransmitters evoked the synthesis of cADPR and the rise of [Ca^2+^]_i_ in neurons and astrocytes of rodent brain, recruiting intracellular CD38 (ARC) [77].

Similar and more complex effects have been observed in the cells of various tissues. In human granulocytes, the activation of P2Y_11_ purinoreceptors by extracellular NAD_o_ was shown to trigger the cascade of interrelated events, including simultaneous generation of IP3, cADPR, cAMP, and bimodal [Ca^2+^]_i_ transients with implication of: (i) PLC-dependent signaling axis (1); (ii) Ca^2+^-activated adenylate cyclase (AC1,8), PKA, and PKA- mediated stimulation of ARC [23].

Activation of signaling axis:

Ca^2+^/AC1,8/cAMP/PKA/ARC/cADPR/RyR/Ca^2+^ (2)

was supposed to be related to Ca^2+^ release from IP3R-dependent Ca^2+^ stores through axis (1). These results demonstrated the possible coupling of P2Y_11_ purinoreceptors to both PLC and AC. 

Recently, experiments performed on murine atrial cells have demonstrated that IP3-mediated Ca^2+^ release may evoke the enhancement of RyR-encoded CaT, recruiting AC1,8 and PKA [78]. However, AC1,8 is thought to be not expressed in murine ventricular cells [78]. While stimulation of P2Y_11_-like receptors in rat ventricular CM induced an increase in intracellular concentrations of Ca^2+^, IP3, cADPR, and cAMP [79], similar to the effects observed in granulocytes [23], indicating the involvement of universal Ca^2+^ signaling mechanisms.

Numerous data have demonstrated that P2Y_11_-like receptors may be coupled both to G_q_ and G_s_-proteins [22,24,25,80], suggesting the recruitment of G_q_/PLC-dependent and Ca^2+^-independent G_s_/AC signaling axes by NAD_o_ in various types of cells including rodent CM [25]. Moreover, exposure of rodent CM and ventricular strips to angiotensin II [81] or β-adrenoreceptors agonists [82,83] was shown to evoke generation of cADPR by intracellular CD38 (ARC) with subsequent RyR-encoded increase of CaT. Presently, CD38, associated with sarcoplasmic reticulum membranes, is considered tightly involved in the control of excitation–contraction coupling and heart hypertrophy [81]. Activation of ARC by PKA is regarded as an important element of β-adrenergic control realized through the signaling axis [75,83]:

G_s_/AC/cAMP/PKA/ARC/cADPR/RyR/Ca^2+^ (3)

However, the distinct signaling pathways linking G-protein coupled receptors with cADPR/RyR-dependent axis remain to be studied insufficiently. The first model of cADPR signaling suggested nitric oxide (NO)-dependent mechanism based on the activation of ARC by protein kinase G (PKG) [84]. Just that time, the indirect effect of PKG on ARC was demonstrated in lymphokine-activated killer cells [85]. Currently, the activation of ARC by PKA is considered as the main element involved in the translation of initial signal from β-adrenoreceptors to RyR in heart cells [71]. However, apparently, both mechanisms are mutually not exclusive. 

Multiscale control, turned on by NAD_o_, could include both the direct fast effect of second messengers (beat-to-beat control) and slowly developing control based on the covalent modification of various target proteins (by PKA, CaMKII, PKG, etc.). In our experiments, the impact of NAD_o_ and ammonia on force F was developed within 15 to 25 min (Figure 1 and Figure 3), suggesting that covalent modification of the channels may be involved in respective mechanisms of excitation–contraction coupling and CaT control. 

### 4.2. NAD_o_ and Ammonia Interplay: Impact of NAD_o_ and Ammonia on Membrane Potential and AP Duration in CM and Contractility of PM

According to published data, NAD_o_ induced hyperpolarization of membrane potential E_r_ and suppressed contractility of GI cells by activating the signaling axis (1) [20]. Apamin prevented the hyperpolarizing effect of NAD_o_ on GI cells, demonstrating that the final targets of this axis were Ca^2+^-activated SK channels [19]. At the same time, the hyperpolarizing effect of NAD_o_ and subsequent suppression of rat striatal neuron firing included the recruitment of both BK and SK channels [26].

#### 4.2.1. Channels Involved in NAD_o_ and Ammonia Antagonism (Interplay)

In our experiments, 4 mM NAD_o_ diminished F_MAX_ of PM by 48–50% (Figure 1a–d), in spite of negligible alterations in RMP and APD of isolated CM (Figure 1e,f). Reactive blue 2 abrogated the NAD_o_-evoked suppression of PM contractility and returned amplitude of force F transients to control values, indicating the involvement of P2Y receptors in the effect of NAD_o_. At this point, we might suggest that, similar to the mechanisms proposed for GI cells contractility control [20], hyperpolarization provided by the activation of Ca^2+^-activated potassium channels via signaling axes (1) and (3) might dominate over the expected positive inotropic effect of cADPR, cAMP and PKA, the key players of axis (3).

At concentrations up to 5 mM, NH_4_Cl did not display any impact on PM contractility, in spite of the depolarization of RMP by ~8 mV and APD shortening by ~20% of isolated CM (Figure 2). However, paradoxically, 5 mM NH_4_Cl abrogated the NAD effect by recovering force F to or over control values (Figure 3a–d), but having a minor impact on RMP and APD90 in CM (Figure 3e,f). Collectively, these results indicate that NAD canceled the effect of ammonia on the electrical functions of CM but reinforced its effect on the contractions of PM. Besides, we may suppose that RMP and APD of CM may be applied with some limitations as a comparison of parameters characterizing NAD/ammonia effects were studied on isolated non-loaded left ventricle CM versus stretched and paced right PM preparations.

Importantly, the antagonism of ammonia and NAD_o_ was not realized in the presence of various channel blockers. Figure 5, Figure 6, Figure 7, Figure 8, Figure 9, Figure 10 and Figure 11 demonstrated that separate blockade of Kir2.x, HCN, LTCC, BK, IK, or SK channels, or inhibition of reverse NCX exchanger abrogated ammonia-induced effect on force F, indicating that all the currents, mediated by respective channels, could be collectively involved in NAD_o_/ammonia antagonism. 

Together, these results might suggest that ammonia, in the presence of NAD_o_, recruits all above-mentioned channels providing coherent cooperation of all the currents involved (I_BK_ + I_IK_ + I_SK_ + IK1_out_ + I_f_ + IK1_in_ + I_NCXrev_ + I_Ca-L_/I_SR_); i.e., it adjusts the interplay of currents involved in RyR-dependent CICR, CaT, and force F control.

#### 4.2.2. LTCC, RyR-Encoded CICR, and cADPR Interplay and Signaling Antagonism

It is well known that LTCC performs the function of spark plugs for RyR clusters engine in CM by switching on CICR that evokes respective CaT and contractions [71,83]. Figure 8 shows that the blockade of LTCC channels strongly suppressed force F and prevented the ammonia effect. The magnitude of RyR-encoded CICR, in turn, depends on the concentration of Ca^2+^ coagonist cADPR synthesized from NAD. In 2000, Hashii et al. demonstrated that [Ca^2+^]_i_ rise, evoked by sustained membrane depolarization of neuroblastoma cells, was strongly modulated by the microinjections of NAD_o_ or cADPR. Depending on the concentrations used, both agonists increased the magnitude of [Ca^2+^]_i_ transients 2 to 3 times, clearly demonstrating LTCC, RyR- evoked CICR and cADPR interplay [86]. Here, we might expect that turning on of signaling axis (3) by NAD_o_, delivering cAMP and cADPR and activating PKA, would activate HCN, LTCC, and RyR-dependent channels with the final rise in CaT. 

However, the suppression of force evoked by NAD_o_ (Figure 1) suggests that the negative hyperpolarizing effect of outward Ca^2+^-activated potassium currents (I_BK_ + I_IK_ + I_SK_) might dominate over the positive impact of second messengers of the axis (3) on LTCC, RyR, and cADPR interplay. Strikingly, ammonia, in the presence of NAD_o_, abrogated this signaling antagonism presumably by recruiting both signaling axes (1) and (3) in coherent control of ECC.

### 4.3. Hypothesis on the Impact of Ammonia on HCN and Kir2.x Channels

In attempts to address this challenge, we suggested that, in the combined application of NAD_o_ and NH_4_Cl, the restoration of contractility evoked by ammonia (Figure 3) may be provided by the activation and coherent cooperation of outward (I_BK_ + I_IK_ + I_SK_ + IK1_out_ + I_NCXrev_) and inward (I_f_ + IK1_in_ + I_Ca-L_/I_SR_) currents, resulting in the accumulation of SR calcium and rise of resting [Ca^2+^]_i_. Further, we assumed that the key drivers of this cooperation are Kir2.x and HCN channels, which are known to be activated by extracellular K^+^_o_ [57,58,59,60,61].

In support of the possible key role of HCN, Kir2.1, and Kir2.2 channels in this effect, we may refer to the results of the experiments with transgenic mice models having overexpressed myocardial LTCC [9] or HCN4 [10] channels. Both models resulted in the shortening of APD, and augmentation of CaT compared to control and suggested rise of [Ca^2+^]_i_. In the first model, the enhancement of I_Ca-L_ amplitude, appearance of persistent Ca^2+^ current, and compensatory rise of several K^+^ currents (including Kir2.2 mediated IK1current) underlie the observed effect, while, in the second model, augmentation of the I_f_ current and the respective rise of [Na^+^]_i_ induced the “reverse mode” operation of NCX (I_NCXrev_) and a three-times rise of CaT magnitude in CM [42]. We suppose that similarly, enhancement of I_f_ and I_NCXrev_ currents could be realized in NAD_o_/ammonia antagonism in our model system.

#### 4.3.1. Potassium, Kir2.x, and HCN Channels, and Positive/Negative Feedback in the System

At this point, we may ask what the mechanism is underlying the overactivation of HCN and Kir2.x channels by ammonia in PM pretreated with NAD_o_. In our opinion, the strict dependence of Kir2.x [57,58,59,60,61] and HCN [62,63,64,65] channels on both voltage and [K^+^]_o_ may underlie this effect and close cooperation of respective currents. Extracellular potassium (rise of [K^+^]_o_) is known to increase the hump-like Kir2.x -mediated outward current IK1_out_, rises the slopes of both inward currents (IK1_in_ and I_f_), and shifts rightward reverse potentials of both currents [57,58,59,60,61,62,63,64,65].

Activation of IK1_out_ by extracellular K^+^ creates a positive feedback loop (PFL) in the chain of currents shaping AP and is implicated in excitation–contraction coupling control. The autocatalytic-like rise of [K^+^]_o_, mediated by IK1_out_, in turn, provides the enhancement of both inward K^+^ currents (I_f_ + IK1_in_) at late phase or repolarization (Phase 3 of AP), creating two negative feedback loops (NFLs) removing K^+^ from interstitial space. A similar mechanism involving PFL/NFL interplay, may underlie the known synergistic effect of outward IK1_out_ and inward I_f_ currents contributing to the mechanisms of automaticity in stem cells-derived CM (“IK1_out_-induced I_f_ activation”) effect observed at phase 3 of AP [6]).

However, initial activation of IK1_out_-dependent PFL is apparently provided by BK + IK + SK channels mediating initial outward hyperpolarizing current (I_BK_ + I_IK_ + I_SK_), delivering K^+^ required for autoactivation of IK1_out_. At this point, we may refer to the known “I_IK_ –induced I_f_ activation” effect, also involved in the control of automaticity in stem cell-derived or ventricular CM with overexpressed SK4 and HCN channels [4,5]. The localization of Kir2.x channels within restricted space of T-tubules [87,88] may provide the reinforcement of IK1_out_-dependent PFL.

Operation of various PFLs in multiscale dynamic systems may bring about oscillations, multistability and triggering phenomena, solitary pulses, trigger waves, etc. [89]. At the moment, there are no known examples demonstrating the operation of formulated above IK1_out_-dependent PFL and delivering similar effects in myocardial cells. However, the example presented below may illustrate the impact of such PFL on neural networks. Extracellular [K^+^]_o_ is known to rise up to 12 mM at focal brain seizures [90] and may evoke biphasic concentration-dependent action on neuronal excitability and seizure activity; at concentrations up to 9 mM potassium prolonged seizure durations and shortened interictal intervals, while, at higher concentrations of 12 mM, potassium blocked seizures events and reversibly, compared to spreading depression, switched on neurons into a depolarization-blocked state implicating I_f_ current mediated by HCN channels [65].

The next question is, what mechanism is providing a critical increase in [K^+^]_o_ at NAD/ammonia antagonism?

#### 4.3.2. Ammonia Evokes Rise of Extracellular Potassium

Hydrated ions NH_4_^+^ and K^+^ have similar ionic radius [33], and this close resemblance between NH_4_^+^ and K^+^ in their effects on various channels and transporters is considered as a key pathogenic factor in acute hyperammonemia [29]. Ammonia, competing with potassium, may increase extracellular concentration of K^+^. At 5–10 mM, NH_4_Cl is known to evoke 1 to 3 mM rise of [K^+^]_o_ in hippocampal slices [37,47] and in situ in mice cortex with craniotomy [91]. In rat models of acute hyperammonemia induced by i.v. infusion of ammonium acetate, a gradual increase of arterial plasma ammonia from 35–40 to 600–650 µM was accompanied by a three-times rise of cortex [K^+^]_o_ up to 11–12 mM, suggesting compromised astrocytes potassium buffering [92]. Similarly, i.p. injections of lethal doses NH_4_Cl induced in coma stage accumulation of arterial plasma concentrations of NH_4_^+^ and K^+^ to 3–4 mM and 11–12 mM, respectively, indicating the systemic effect of ammonia on various types of cells, not restricted by the impairment of astrocyte K^+^ control [93]. 

#### 4.3.3. Suggested “Twin-Brother’s Effect” of Potassium and Ammonia on HCN and Kir2.x Channels Interplay 

Here, we might suppose that the rise of resting [K^+^]_o_ to some critical level, evoked by ammonia, may underlie the activation of both IK1_out_-dependent PFL and I_f_ + IK1_in_ -dependent NFLs providing IK1_out_/(I_f_ + IK1_in_) interplay. We also may hypothesize that activation of both types of channels (Kir2.x and HCN) by potassium may be reinforced by NH_4_^+^, having close resemblance to K^+^ [33], i.e., by its “twin brother’s effect” on IK1_out_/(I_f_ + IK1_in_) interplay (i.e., PFL/NFLs interplay). 

Note that 5 mM NH_4_Cl, by itself, did not induce substantial alterations in force F of PM, in spite of marked depolarization of RMP (Figure 2). While, in the cells pretreated with NAD_o_, ammonia, in spite of minor alterations of RMP and APD in CM, upraised force F to or over control values (Figure 3), indicating that the impact of ammonia (or potassium + ammonia) on Kir2.x and HCN channels interplay may depend on cAMP and PKA, the elements of signaling axis (3). 

Figure 4 shows steady-state current–voltage (I/V) relationships that are predominantly mediated by currents associated with the activity of were mostly mediated by Kir2.x, HCN, either-go-go and two-pore-domain (K2P) potassium channels. Figure 4a demonstrates that 5mM NH_4_Cl significantly potentiated the inward rectifying fraction of integral ‘steady-state’ current with a substantial rightward shift of Er resembling IK1_in_ and I_f_ currents. These changes were accompanied by a rightward shift in the potential for the maximal current density of hump-like outward current resembling, apparently, IK1_out_ current (creating PFL, red line). Importantly, this effect of ammonia has some qualitative similarities to the effect of potassium on I/V relationship (Figure 4d) supporting the notion on “twin-brother’s effect” of ammonia. Weak hump-like effects of potassium (and ammonium) ions may be explained by the fact that K^+^ evoked much more strong activation of peak compared to sustained currents mediated by Kir2.x channels [58]. Here, we have registered steady-state (sustained) net current.

Collectively, we might suggest that being evoked by the ammonia rise of [K^+^]_o_ and switching on of signaling axis (3) may underlie NAD_o_/ammonia antagonism and coherent outward/inward currents interplay.

### 4.4. Working Hypothesis of NAD/Ammonia Antagonism

**Figure 12:**
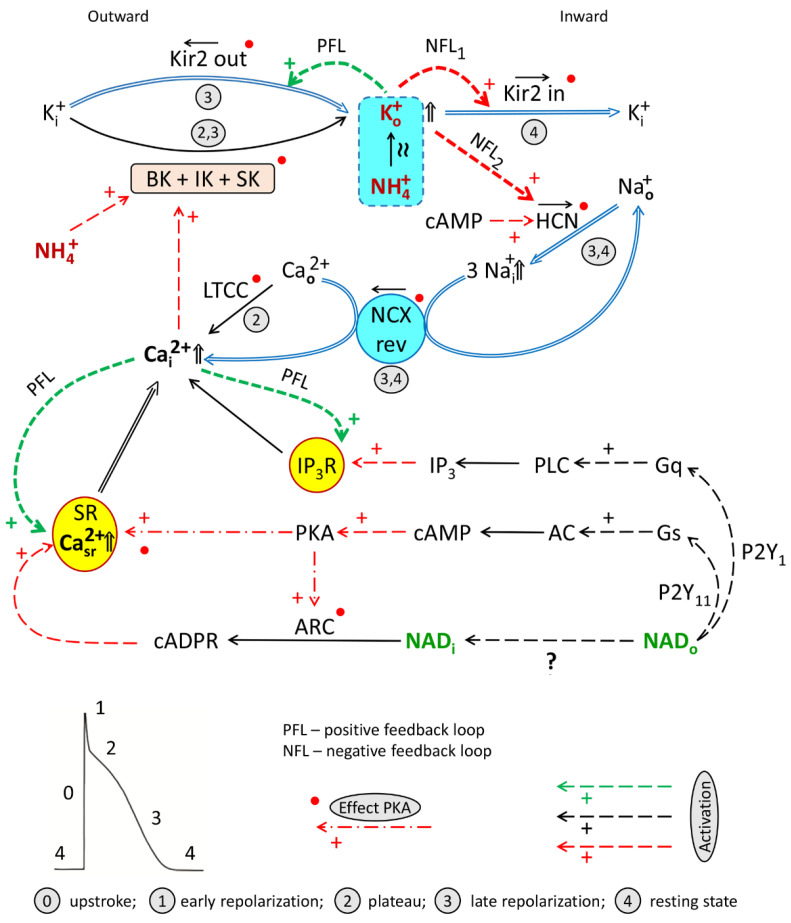
The mechanistic model describing ion channels and second messengers implicated in the control of rat PM contraction under NAD and ammonia antagonism (interplay). The model includes: signaling axes (1) and (3) turned on by NAD_o_ and acting through P2Y_1_ and P2Y_11_ receptors, respectively, and delivering second messengers IP3, cAMP, and cADPR; operation of BK+IK+SK+Ki2.x channels mediating hyperpolarizing outward K^+^ currents and delivering K^+^ required for activation of Kir2.x and HCN channels; IK1_out_-dependent positive feedback (PFL) based on the activation of Kir2.x channels by K_o_^+^, providing K_o_^+^-induced K_o_^+^ rise (dark green dotted arrow); shown by red dotted arrows two negative feedbacks (NFLs), i.e., K_o_^+^-induced K_o_^+^ removal based on the activation by K_o_^+^ of HCN and Kir2.x channels mediating depolarizing I_f_ +IK1_in_ net current; I_f_ current-evoked rise of [Na^+^]_i_ inducing reverse mode operation of NCX and accumulation of [Ca^2+^]_i_ with final augmentation of LTCC/cAPDR/RyR interplay determining magnitude of CaT. In this model, a light blue oval depicts NH_4_^+^-induced rise of [K^+^]_o_ and a possible “twin-brother’s effect” of ammonia and potassium on Kir2.x and HCN channels. The activation of BK, IK, and SK channels by Ca^2+^ and BK channels by NH_4_^+^ is shown in left top part of the cartoon by red dotted arrows. Activation of various channels by PKA phosphorylation is shown by brown dotted arrows and red rounds. The possible effect of CaMKII is omitted for simplicity. RyR and IP3R-encoded CICR (creating two PFLs) and activation of these channels by the coagonists cADPR and IP3, respectively, are shown as purple curved arrows. Figures in gray circles indicate 5 phases of AP [11] including upstroke (0), early repolarization (1), plateau (2), late repolarization (3), and resting state (4).

Overall, based on known literature data analysis and our results of inhibitory analysis, we may formulate several assumptions providing the ground for the mechanistic model describing outward/inward currents interplay presumably taking place at NAD_o_/ammonia antagonism in PM. These assumptions are presented in Figure 12 and can be formulated as follow:

1. Signaling axes (1) and (3) turned on by NAD_o_ and acting through P2Y1 and P2Y11 receptors, respectively, and delivering second messengers IP3, cAMP, required for the activation of HCN channels, and cADPR, involved in LTCC/RyR-dependent CICR/cADPR interplay and CaT rise.
2. Cooperation of BK + IK + SK channels. Activation by calcium of BK, IK, and SK channels mediating hyperpolarizing outward K^+^ current delivering K_o_^+^ required, in turn, for activation of Kir2.x and HCN channels and forming PFL and NFLs;
3. PFL/NFLs interplay: sequential turning on of (i) IK1_out_-dependent hyperpolarizing PFL based on K^+^_o_-induced K^+^_o_ rise and (ii) two NFLs (K^+^_o_-induced K^+^_o_ and Na^+^_o_ removal) mediating depolarizing I_f_ + IK1_in_ net current based on the activation by K_o_^+^ of HCN and Kir2.x channels.
4. “Twin-brother’s effect”, based on suggested NH_4_^+^-induced rise of [K^+^]_o_ and overactivation of Kir2.x and HCN channels by both these ions.
5. [Na^+^]_i_/[Ca^2+^]_i_ rise: K^+^_o_ and cAMP-dependent augmentation of the If current providing the rise of [Na^+^]_i_ and reverse mode operation of NCX (NCXrev) resulting in the accumulation of [Ca^2+^]_i_ and augmentation of LTCC/cAPDR/RyR interplay determining the magnitude of CaT.
6. Activation of BK channels by NH_4_^+^.
7. Activation of various channels by PKA and CaMKII phosphorylation.

Collectively, we might suggest that in the absence of ammonia, the negative hyperpolarizing effect of outward Ca^2+^-activated potassium currents (I_BK_ + I_IK_ + I_SK_) may dominate over the positive impact of second messengers of the axis (3) on LTCC, RyR, and cADPR interplay, providing the suppression of PM contractions evoked by NAD_o_ (Figure 1). While, in the presence of NAD_o_ (i.e., of axis (3)), ammonia abrogated this signaling antagonism (Figure 3) presumably by increasing extracellular K^+^ (K^+^ + NH_4_^+^) to concentrations sufficient for overactivation of Kir2.x and HCN channels and reinforcement of PFL/NFLs interplay.

It is critical to mention that these assumptions would require further investigation through dissection of every suggested pathway involved in the regulation of cardiac contractility by NAD_o_/ammonia antagonism both at experimental and modeling levels.

### 4.5. On the Dormancy of SK, BK, and HCN Channels in Ventricular CM

Numerous studies suggest that SK, BK, and HCN channels, being dormant at control, are upregulated in the ventricular myocardium at increased adrenergic drive, during the remodeling of ischemia, hypertrophy, or heart failure. Application of RT-PCR, Western blot, and immunochemistry indicate expression in rodent ventricular myocytes of SK [3,94,95], BK [96,97], and HCH [98,99,100] channels. Some data indicate that at control conditions, only up to 16% of ventricular myocytes express HCN channels, while in ventricular hypertrophy, the number of I_f_ positive cells may rise to 46% [100]. Similarly, covalent modification with CaMKII [94] or PKA [3,95] is considered to be important for the activation of SK channels in ventricular CM and the appearance of the visible effect of apamin. Here, we suppose that similar effects with HCN, BK, and SK channels “awakening” may be realized at NAD/ammonia antagonism owing to the activation of PKA by the P2Y receptors-dependent second messenger signaling axis (3).

### 4.6. Prospects and Clinical Relevance

Numerous data, based on pharmacological manipulations, indicate possible implications of HCN, Kir 2.x, and Ca^2+^-activated potassium channels in the pathological processes related to brain focal seizures and spreading depression [64,65,101] and cardiovascular ischemia/reperfusion events as atrial and ventricular arrhythmogenic activity and fibrosis [102,103,104,105,106]. To date, we might suggest that the clusters of respective channels, organized as positive/negative feedback units (systems), may be prospective pharmacological targets for the treatment of respective diseases.

### 4.7. Limitations of the Study

The present work examined the mechanisms underlying NAD/ammonia antagonism realized in rat PM and disclosed the implication of several ion channels in the mechanisms, including HCN and Kir2.x channels mediated currents forming a core element of the system with PFL/NFLs interplay. Strong dependence of outward/inward rectification for both types of channels on extracellular K^+^ underlies the formation of respective PFL and NFLs. However, it is well known that the rectification of hERG-mediated current (IKr) is also strongly dependent on [K^+^]_o_ [107,108]. Therefore, IKr-dependent PFL might also contribute to discussed PFL/NFLs and NAD/ammonia antagonism. 

Second, limitations stem from the lack of studies on the contribution of CaMKII in NAD/ammonia antagonism, especially at the excess of [Ca^2+^]_i_. At a moment, we consider IKr-based PFL and the effects of CAMKII on numerous targets as secondary mechanisms related to sufficient (not necessary) conditions. However, these limitations should be overcome in the future.

## 5. Conclusions

We suggest that promoted by NADo switching on of signaling axis (3) delivering second messengers cAMP, cADPR, and Ca^2+^ and activating PKA—represent necessary conditions for NAD/ammonia antagonism and IK1_out_/(I_f_ + IK1_in_) currents interplay. While sufficient conditions might be brought about by NH_4_^+^ evoked increase of [K^+^]_o_ to critical concentrations, required for overactivation of Kir2.x and HCN channels by K_o_^+^ (and NH_4_^+^), with resulting increase of [Na^+^]_i_/[Ca^2+^]_i_, and restoration force of PM contractions.

## Data Availability

The datasets generated during the current study are available from the corresponding author upon reasonable request.
